# Estimated Secondary Structure Propensities within V1/V2 Region of HIV gp120 Are an Important Global Antibody Neutralization Sensitivity Determinant

**DOI:** 10.1371/journal.pone.0094002

**Published:** 2014-04-04

**Authors:** Maxim Totrov

**Affiliations:** Molsoft LLC, San Diego, California, United States of America; Meharry Medical College, United States of America

## Abstract

**Background:**

Neutralization sensitivity of HIV-1 virus to antibodies and anti-sera varies greatly between the isolates. Significant role of V1/V2 domain as a global neutralization sensitivity regulator has been suggested. Recent X-ray structures revealed presence of well-defined tertiary structure within this domain but also demonstrated partial disorder and conformational heterogeneity.

**Methods:**

Correlations of neutralization sensitivity with the conformational propensities for beta-strand and alpha-helix formation over the entire folded V1/V2 domain as well as within sliding 5-residue window were investigated. Analysis was based on a set of neutralization data for 106 HIV isolates for which consistent neutralization sensitivity measurements against multiple pools of human immune sera have been previously reported.

**Results:**

Significant correlation between beta-sheet formation propensity of the folded segments of V1/V2 domain and neutralization sensitivity was observed. Strongest correlation peaks localized to the beta-strands B and C. Correlation persisted when subsets of HIV isolates belonging to clades B, C and circulating recombinant form BC where analyzed individually or in combinations.

**Conclusions:**

Observed correlations suggest that stability of the beta-sheet structure and/or degree of structural disorder in the V1/V2 domain is an important determinant of the global neutralization sensitivity of HIV-1 virus. While specific mechanism is to yet to be investigated, plausible hypothesis is that less ordered V1/V2s may have stronger masking effect on various neutralizing epitopes, perhaps effectively occupying larger volume and thereby occluding antibody access.

## Background

Neutralization by antibodies, along with cellular immunity, is a key defense mechanism against viral infection. Most clinical isolates of HIV-1 virus are notoriously difficult to neutralize by antibodies. This resistance is contributing to both, the inability of human immune system to control HIV infection in the vast majority of individuals and the fact that despite decades of concerted efforts to create an effective prophylactic HIV vaccine, only a rather limited success has been reported so far (vaccine trial RV144 in Thailand) [1]. Apart from the common viral resistance mechanisms of evasion via frequent mutations, HIV appears to have evolved highly efficient ways of ‘hiding’ vulnerable conserved immunogenic structures. The only viral proteins exposed on the HIV particles are the envelope glycoprotein (‘env’) gp120/gp41 trimeric spikes which mediate host cell attachment and fusion [2]. The spikes contain conserved interfaces and other structures that are necessary for receptor (CD4) [3] and co-receptor (CCR5 or CXCR4) binding [4] and eventual fusion. However, the virus appears to disguise these vulnerable targets from the host's immune system under a heavy glycosylation layer [5], behind highly variable elements [6], within narrow crevasses of the structure that are poorly accessible to antibodies, and using other mechanisms of epitope ‘masking’ [7] that are still poorly understood.

Yet this resistance varies greatly between different virus isolates, and a ‘Tier’ system has been proposed to classify HIV strains and to provide a virus panel for objective evaluation of immune sera and monoclonal antibodies in terms of their neutralization potency. Importantly, strains that resist neutralization often do so across multiple antibody types targeting different epitopes.

In principle, neutralization resistance variations should be determined by env sequence and ultimately by the structure and dynamics of the spike. It has been proposed that ‘intrinsic reactivity’ of the *env* trimer, i.e. its propensity to undergo conformational transition to lower-energy states from the initial native state, provides an important contribution to global inhibition sensitivity [8]. However, no general sequence-structure-function (i.e. resistance) relationships have been established so far, although singular mutations that dramatically alter resistance have been reported [5], [9], [10].

Intriguingly, it was demonstrated that V1/V2 region of gp120 is an important determinant of the overall neutralization sensitivity of the HIV-1: modifications and deletions often increase neutralization sensitivity [6], [11], and swapping the V1/V2 sequence of a neutralization-sensitive virus for a V1/V2 from a resistant one conferred neutralization-resistant phenotype, and conversely [12], [13]. Binding experiments and mathematical modeling allowed dissection of V1/V2 masking effects on the V3 loop [14]. Some controversy exist as to whether V1/V2 and V3 interactions are inter- or intra- protomer: mathematical modeling approach indicates interactions in trans (i.e. between neighboring subunits) [14] while different mixed trimer expression experiments suggest that V3 masking occurs within each protomer (in cis) rather than between protomers [15]. Possibly both mechanisms coexist [16].

**Figure 1:**
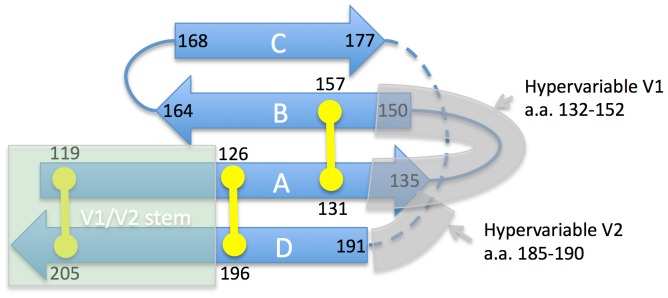
Schematic secondary and tertiary structure organization of the V1/V2 domain in HIV gp120. Disulfide bridges are shown in yellow. Beta-strands(shown as block arrows) are assigned according to the X-ray structure (PDB ID 3U4E). In this structure stem region is replaced by an unrelated scaffold, but the stem is independently observed to form anti-parallel strands in multiple gp120 X-ray structures (e.g. PDB ID 2B4C). Dashed line indicates polypeptide chain segment unresolved in the available X-rays although N-terminal part of it has well-conserved sequence, including the integrin binding motif.

**Figure 2:**
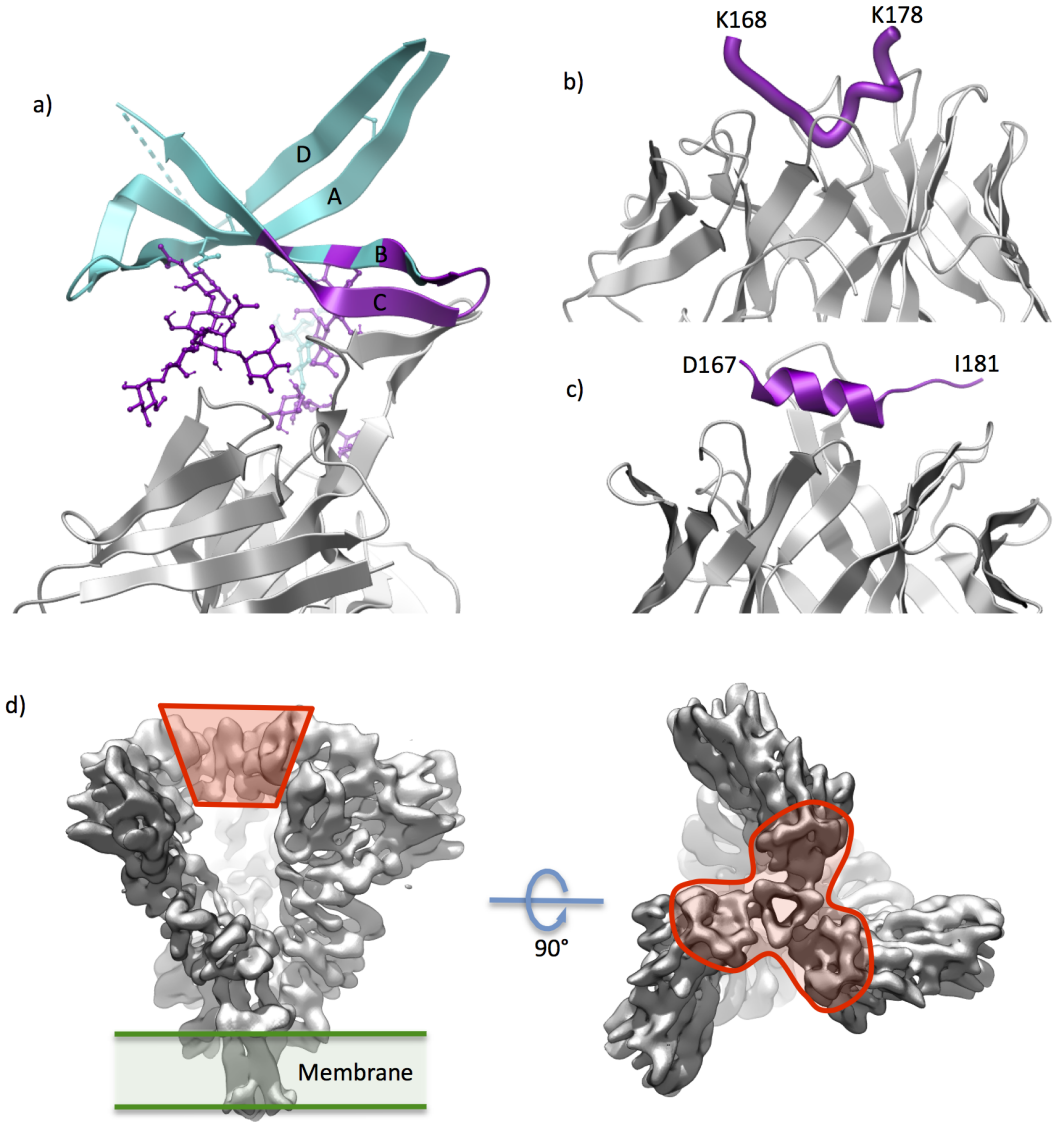
Available X-ray structures of V1/V2 domain and its fragments. (a) Complex of the V1/V2 domain (blue ribbon, glycans shown in ball-and-stick representation; parts interacting with the FAb are in magenta) grafted on a scaffold protein (not shown) and the BNAb PG9 (grey ribbon). (b) and (c) V1/V2 fragments (magenta) in complex with mAb CH58 and CH59, respectively (grey ribbon). Structures were visualized from Protein Data Bank entries 3U4E, 4HPO and 4HPY. (d) Visualization of EM electron density map (EMDataBank, www.emdatabank.org, accession no. EMD-5447) of the trimeric env spike. Approximate localization of the V1/V2/V3 loops (forming presumed ‘TAD’) is delineated in red.

Until recently, little has been known about the structure of V1/V2 domain and the two segments in it delineated by disulfide bridges were viewed as ‘loops.’ V1/V2 received limited attention in vaccine development efforts because of its high variability and apparent limited functional importance – V1/V2 deleted virus often remains replication competent [17]. The region was truncated out of all gp120 ‘core’ structures solved by X-ray crystallography to date. The interest in the region soared when broadly neutralizing antibodies targeting V1/V2 were reported [18] and soon thereafter crystal structure of V1/V2 domain was solved in complex with broadly neutralizing monoclonal antibody (BNAb) PG9 [19]. In this structure, V1/V2 domain was grafted on an unrelated scaffold protein. The X-ray structure established that V1/V2 domain is organized into a compact 4-strand anti-parallel beta-sheet fold (Fig 1). The antibody was observed to interact primarily with strand ‘C’ and glycans (Fig. 2a). Subsequently solved structure of the closely related BNAb PG16 complex exhibits a very similar binding mode but more extensive glycan interactions [20]. Next, two complexes of antibodies (CH58 and CH59) with linear epitope peptide fragments of V1/V2 were solved [21]. Low resolution electron microscopy (EM) structure provided first unambiguous data on the localization of V1/V2 domain within the trimeric gp120 spike [22]. Certain EM structures suggest that V1/V2 and V3 loops form a ‘trimer association domain’ (TAD) near the apex of the gp120 spike (Figure 2d) [23]. Most recently, first medium-resolution trimeric X-ray structure of the so-called SOSIP gp140 has been reported [24], and the V1/V2 conformation in context of this trimer corresponded well to that observed in PG9/PG16 complexes,

Antibody responses to V1/V2 in the sera correlated with protection in the RV144 vaccine trial in Thailand [25], the only HIV vaccine trial that demonstrated (limited) efficacy [1]. Antibodies induced by the vaccine targeted conserved mid-region of the V2 loop [26]. Most recently, ‘sieve’ analysis demonstrated that substitutions at two positions in V1/V2 correlate strongly with susceptibility of the virus to vaccine protection in RV144 trial [27]. Immunodominant site on V2 was revealed by epitope mapping of conformational V2-specific human antibodies [28], and epitopes of 8 mouse antibodies were localized to the C-strand termini [29]. Additionally, polyclonal antibodies in a broadly neutralizing blood serum from an AIDS patient (coded CAP256) were shown to target central residues of the BC hairpin [30].

Remarkably, linear epitopes of the mAbs CH58 and CH59, which localize to the stretch of the V1/V2 sequence corresponding to the C-strand in mAb PG9 complexes, are structurally distinct: CH58 binds a largely alpha-helical conformation of the antigen, while in CH59 complex it is a coil with elements of 3–10 helical and extended conformations (Figs. 2b and 2c).

Can structural variability of V1/V2 domain underlie its influence on the neutralization sensitivity (NS) of the HIV strains? In the absence of the structural data for a range of V1/V2 domains from different strains and in the native context, it may still be possible to tease out certain structural correlates from simple sequence-based biophysical descriptors. Since the possibility of secondary structure switch between beta-strand, coil and/or alpha helix is indicated by the X-ray structures, propensities to form alpha-helix and beta-strand are of particular interest. While all 20 naturally occurring amino-acids are found within all types of secondary structures, preferences towards one or another structure that vary significantly between the amino-acids are well-established [31], [32], both via statistical analysis of protein structures [33] as well as via energy calculations [34] and measurements [35]. Accordingly, a number of beta-strand (alpha-helix) propensity scales have been proposed, attributing an energy cost/gain of having a particular amino-acid type within a strand (or helix), usually relative to alanine [36], [37]. Total propensity calculated over a stretch of polypeptide sequence can provide a measure of its preference for a particular secondary structure state. Herein, an investigation into correlations of such propensities for V1/V2 loop domain of gp120 and certain segments within it with virus sensitivity to antibody neutralization is presented.

## Results and Discussion

### Overall beta-sheet propensity of V1/V2 domain correlates with neutralization sensitivity

**Figure 3:**
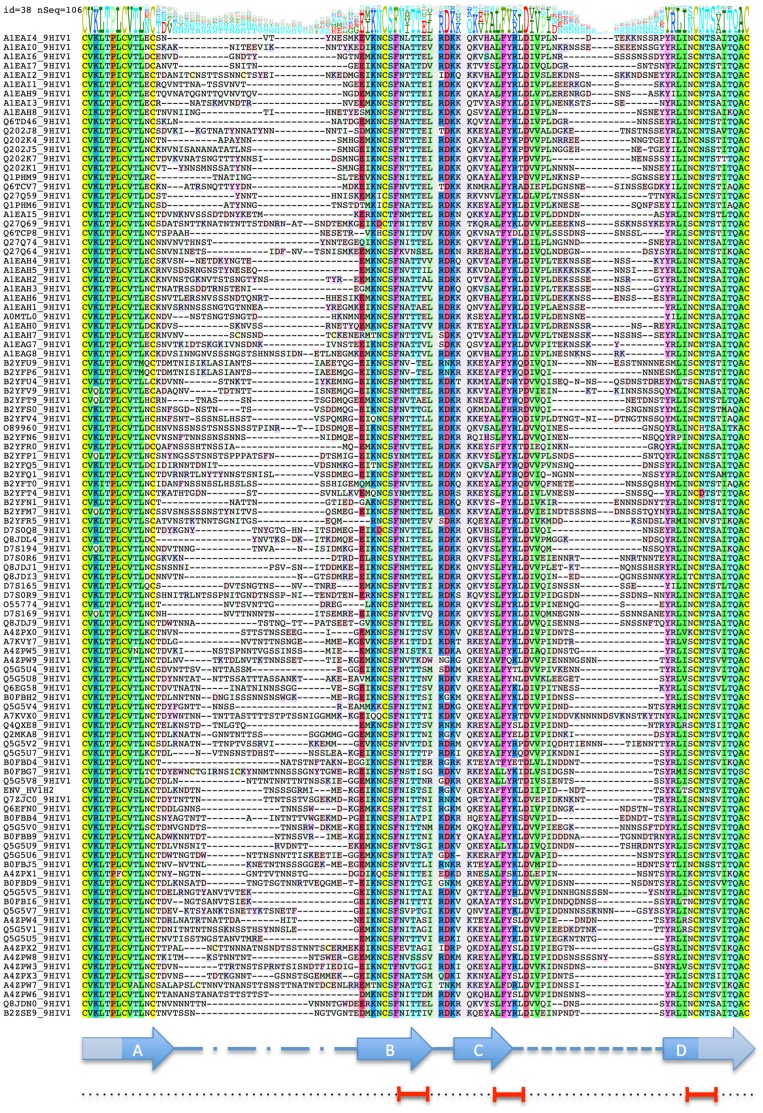
Multiple alignment of 106 sequences of V1/V2 domains in HIV gp120. Sequence profile is shown above the alignment: stack of amino-acid residue letter codes above each position indicates frequencies (taller letters – more frequent), with most frequent one at the bottom of the stack. Secondary structure cartoon is shown below the alignment as assigned in the X-ray structure (PDB 3U4E). Also indicated at the bottom are the three 5-residue segments (red intervals) that represent BSP/NS ‘hotspots’.

Correlation of the neutralization sensitivity for 106 HIV isolates on the tiered neutralization assessment panel [38] with structural propensities of the V1/V2 domain was analyzed. Total beta-sheet propensity (BSP) across 60 well-aligned amino-acid positions (Fig. 3) within the V1/V2 domain and its stem correlated with the log_10_ of neutralization ID50 (50% inhibitory dose) by HIVIG (HIV immune globulins). Pierson correlation coefficient R was 0.34. While modest, correlation was highly statistically significant with the p-value estimate of 0.0003. For comparison, when the same calculation was performed for alpha-helical propensity, R = −0.14 with p-value estimate of 0.15 was obtained.

### Localization of BSP/neutralization sensitivity correlation ‘hotspots’ within V1/V2 domain

**Figure 4:**
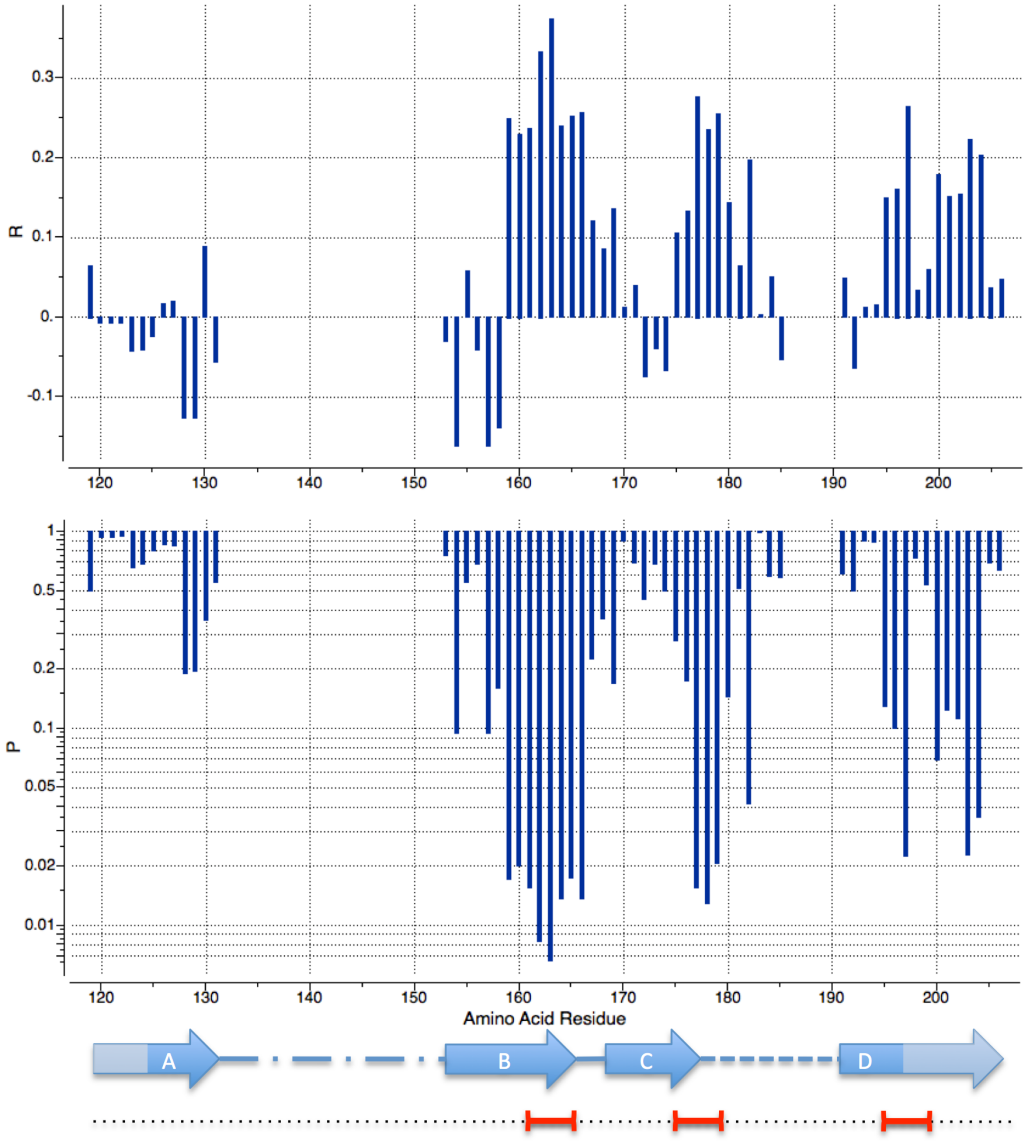
Plots of Pierson correlation coefficients R and p-values for the BSP/NS correlation (top and middle, respectively). BSPs are calculated for 5 amino-acid segments centered on each position within the three conserved stretches of V1/V2 and its stem. Gaps in the plot correspond to the two hyper-variable regions that aligned poorly and were excluded from the analysis (see also Fig. 3). Also shown are the secondary structure and three ‘hotspot’ segments (see legend of Fig. 3).

To investigate whether there were particular regions within V1/V2 where the correlation was localized, total propensities within a sliding window of 5 amino-acids around each position were calculated and correlations with log_10_ ID50 evaluated. Short 5-residue segments rather than individual residues were analyzed because secondary structure formation is a cooperative process and therefore total propensities within a window are expected to be more structurally meaningful and less noisy. For 15 positions p-values of the correlations reached statistical significance (p<0.05). Importantly all of them had the same (positive) sign of the correlation, i.e. lower ID50s correlated with more negative propensity (i.e. more stable beta-strand structures). When plotted along the amino-acid sequence, two peaks of logID50/beta propensity correlation centered at positions T163 and Y177 were observed (p-values of 0.007 and 0.01), as well as a weaker peak at S197 (p = 0.02)(Figure 4). Notably, the last segment extends beyond the end of V2 proper into V1/V2 stem. For comparison, same analysis was performed with alpha-helix propensities. Weaker correlations with oscillating sign were observed and only 4 segments (centered at L130,C131,P183 and A200) reached p-values somewhat below 0.05 cutoff (see Figure S1).

Remarkably, the two pronounced peaks of beta-strand propensity correlation corresponded well to the C-terminal parts of adjacent beta-strands (B and C) observed in the available X-ray structures (Protein Data Bank IDs 3U4E and 3U2S). It is therefore plausible that propensities from both segments combine to determine the overall stability of the beta-hairpin structure formed by the two strands, perhaps regulating the length of the stably folded part versus less ordered connecting loops. Indeed, total BSP from the central 32-residue V1/V2 segment (E153-I184) showed Pierson correlation coefficient with neutralization log_10_ ID50 of 0.35, similar to that of the total BSP of the entire conserved V1/V2 domain above, and p-value of 0.00002 – actually better than that for the entire domain. Third weaker peak was in the middle of the strand D, extending into V1/V2 stem.

Stronger correlation could be achieved if contributions from the top three independent 5 residue segments were combined (I161-I165, F175-L179 and S195-S199), with R = 0.47 (confidence interval 0.35–0.57) and p<0.000001 (p-value for the correlation here is not strictly meaningful since the choice of contributing segments is done *a posteriori*). The correlation plot log_10_ ID50/ΔG_BSP(I161-I165, F175-L179, S195-S199)_ is shown on Fig 3. On the other hand, no detectable signal is seen in N-terminal strand A. We can hypothesize that this strand is more stable, possibly due to two disulfide bridges, and does not experience significant structural variability.

### Persistence of the correlation within different clades of HIV

Robustness of the correlation was investigated by partitioning the data into subsets according to virus clades: the 106 viruses panel contained 40 clade B, 24 clade C, 11 in CRF (circulating recombinant form) BC and 11 in CRF AG. Other clades and forms had only a few representatives each and were combined in a fourth group of 20 viruses, mostly related to clade A. Significant correlation was still present in the first two groups, with strongest correlation in clade B, and somewhat weaker in clade C (Table 1). It should be noted that to reach statistical significance (p-value<0.05) at expected R∼0.4 the dataset generally needs to have >18 points [39]. Thus CRF BC also had comparable R value but statistical significance could not be reached likely due to the smaller size of the group. Correlation values were in the same range for combinations of these three groups, indicating that underlying relationship holds within and across the two clades and their CRF. Thus observed correlation did not arise from possible trivial co-variations with sequence and neutralization sensitivity differences between clades. On the other hand, CRF AG and the fourth group that combined other clades, exhibited only weak, statistically non-significant correlation. It is possible that neutralization sensitivity differences unrelated to the V1/V2 structural effects obscure the correlation in this genetically diverse group.

**Table 1:** BSP/neutralization sensitivity correlations within clade subgroups of the HIV virus panel.

Clade	Number of viruses	R (p-value)
All	106	0.47 (<0.000001)
B	40	0.48 (0.002)
C	24	0.4 (0.05)
CRF BC	11	0.4 (0.2)
B + C	64	0.5 (0.000005)
B + C + CRF BC	75	0.51 (<0.000001)
CRF AG	11	0.25 (0.4)
All other (mostly A and related)	20	0.2 (0.4)

## Conclusions

Conformational heterogeneity of V1/V2 region was recently demonstrated by the X-ray investigations of PG9/PG16, CH58 and CH59 mAb complexes with their epitopes: the C-strand of PG9 complex that encompasses the key residues involved in binding to all three mAbs exhibits beta-strand secondary structure in the first case, mostly alpha-helical secondary structure in the second case, and coiled conformation in the third. While this ability to adopt different conformations was so far directly demonstrated only in antibody binding, it may also have a functional role, i.e. the conformation may change during the transitions associated with attachment and fusion, and prevalence of different conformations may also vary from virus strain to virus strain or from clade to clade. Herein it is demonstrated that a simple sequence-based measure of the propensity to form beta-structure within B/C hairpin region of the V1/V2 domain significantly correlates with neutralization sensitivity of the virus.

Modulation of this propensity likely either triggers switch from one conformation to another or affects equilibrium between multiple conformations that the domain can adopt. Another possibility is that beta-sheet propensity controls order/disorder transition or equilibrium within V1/V2 domain. Functional importance of intrinsic disorder in proteins [40] and more specifically viral proteins [41], [42] is increasingly gaining recognition. A range of mechanisms by which the conformational changes in the V1/V2 domain affect neutralization sensitivity can be proposed. Recent low-resolution structural studies of the gp120 trimers indicate that V1/V2 domains localize near the axis of the spike and therefore the three domains likely contact and interact with each other. These interactions may influence overall configuration of the trimer, making it more ‘open’ or ‘closed’ and modulating accessibility of multiple unrelated epitopes. On the other hand, parts of loops within V1/V2 domain and their glycan decorations may extend over other immunogenic regions of gp120 and shield them from antibody access. V3 region in particular has been shown to be subject to ‘masking’ effects that are largely V1/V2 mediated [14]–[16], [43]. We can hypothesize that less-ordered V1/V2 domain may be effectively bulkier and block access to various other epitopes stronger than when it is tightly folded. Finally, V1/V2 in itself is an important neutralizing Ab target and some of the observed changes in neutralization sensitivity to immune serum may simply reflect differences in presentation of the intrinsic V1/V2 neutralizing epitopes.

**Figure 5:**
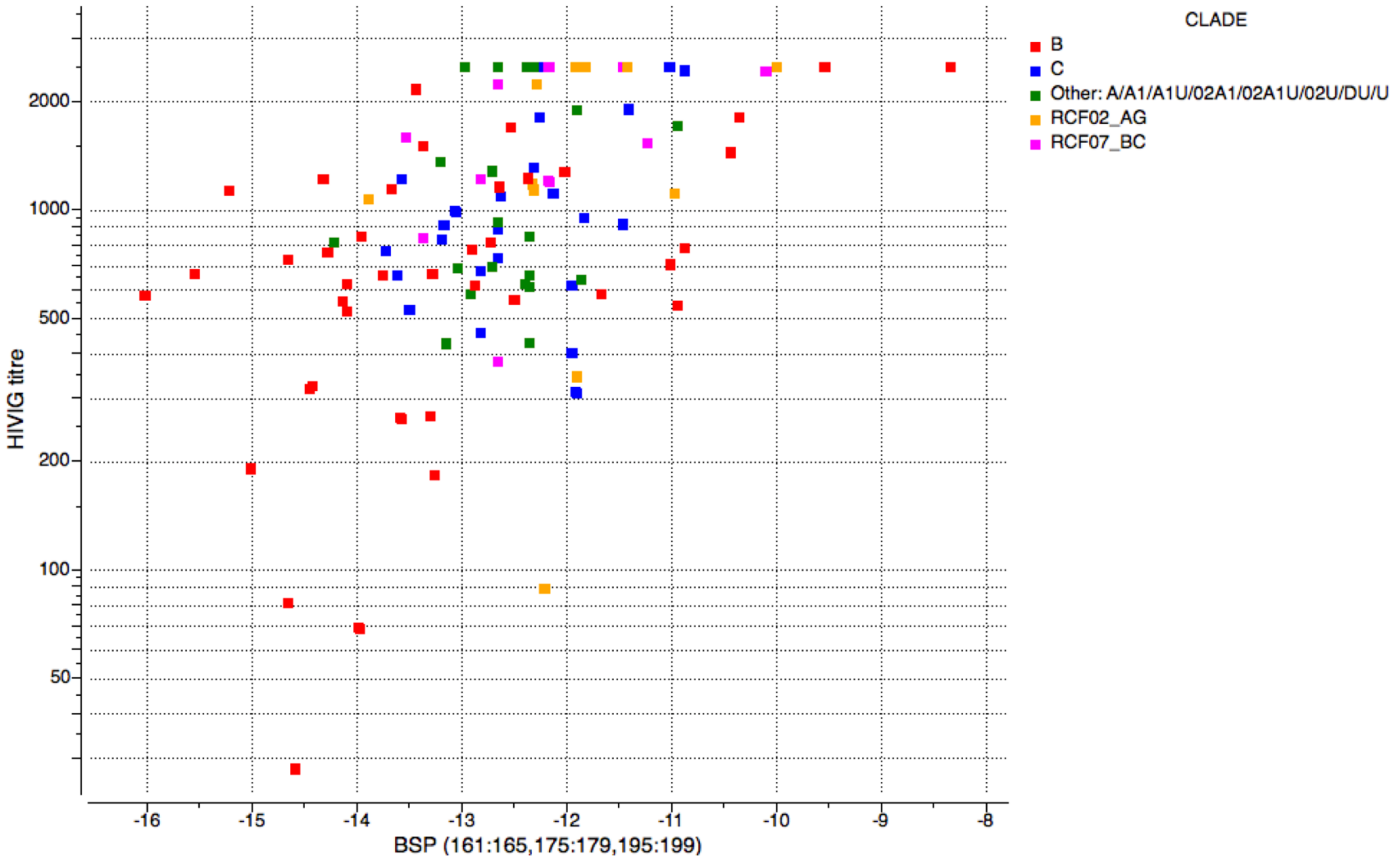
Correlation plot of HIVIG ID50 versus the total BSP calculated across the three ‘correlation hotspot’ segments (15 positions, amino-acids 161:165,175:179 and 195:199).

Clearly, the described effect of V1/V2 beta-strand propensity is not the only factor that determines the neutralization resistance of HIV virus, as the wide spread of the correlation plot (Fig. 5) indicates. It has been reported that a single position mutation D179N could convert highly neutralization resistant virus into a sensitive one [9]. Presence or absence of glycans at certain positions was also shown to play a role [5]. Mutations outside V1/V2 have been demonstrated to affect global sensitivity as well [10]. Nevertheless, all previously reported mutation data was of singular nature without a clear common trend. Generality of the observed effect of secondary structure propensity points towards a common conformational mechanism of neutralization sensitivity modulation applicable to many HIV virus strains. To establish how local secondary structure preferences affect global neutralization sensitivity further structural investigations of V1/V2 region in relation to different strains of HIV will be needed. The finding may have implications for HIV vaccine design, which increasingly incorporates structural considerations.

## Methods

### Sequences and alignment

Gp120 sequences of 106 HIV isolates on the tiered neutralization assessment panel [38] were extracted from the GeneBank. The panel contains 109 viruses, however the three ‘Tier 1A’ viruses (MW965.26, SF162.LS and MN/H9) were excluded as clear outliers with neutralization titers orders of magnitude outside the general range. Sequences were aligned and the V1/V2 region sub-alignment extracted in ICM [44], [45] (Fig. 3). Two segments of V1/V2 domain, amino-acids (AA) T132-G152 and D185-S190 (numbering and AA residues here and throughout the paper follow the *env* sequence of HIV1 strain HXB2) are extremely variable both in composition and length. They were excluded from the analysis. Also excluded were two short inserts present only in one sequence each (4 AAs between positions 169/170 in H078.14 and 1 AA between positions 165/166 in 9021.14.B2.4571). Because X-ray structures of V1/V2 domain show that C- and N- terminal secondary structure elements (beta strands A and D) extend through the C126-C196 disulfide bridge that formally separates V1/V2 domain itself and the so-called ‘stem’ (independently observed forming beta-strand in other gp120 X-ray structures), sequence segments belonging to the stem were also included in the analysis (up to the next disulfide C119-C205). As seen in the results, only minor signal was observed outside the V1/V2 domain proper.

### Neutralization data

As a measure of neutralization sensitivity (NS) of the virus, neutralization data from ref [38] was used and represented log10 ID50 titers of HIVIG (HIV immune globulin) in μg/ml, thus higher titer numbers corresponded to lower sensitivity. HIVIG is a purified HIV+ Ig reagent that is obtained from the NIH AIDS Research and Reference Reagent Program. HIVIG is prepared from pooled plasma of asymptomatic, HIV antibody positive donors. Titer values varied from 28 to >2500 μg/ml (the titers exceeding 2500 were not measured precisely, only the censored value is available). Three excluded ‘Tier 1A’ strains (see above) had titers of <0.02, 6 and 7 μg/ml. Log of the HIVIG concentration rather than the concentration itself was used to calculate correlations with structural propensities. because logarithm of concentration should linearly relate to the binding free energy. Structural propensities reflect free energy involved in secondary structure formation, thus physically homogeneous parameters of the system were being correlated.

### Structural propensities

Smith, Withka & Reagan [36] scale of beta-sheet propensities was used. The scale is based on protein stability changes upon Ala/Xxx substitutions, experimentally measured via melting temperature. This scale was chosen because the ΔΔG (kcal/mol) values are expected to directly reflect changes in stability of beta-sheets associated with a substitution. Pace & Scholtz [37] scale was used for alpha-helix propensity.

Initially, total beta-sheet propensity over the conserved elements of V1/V2 domain were evaluated for each *env* sequence:
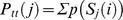
where *S_j_(i)* is the amino acid at i-th position in j-th sequence according to the alignment, *p*() is the appropriate propensity according to the scale and summation is over all well-aligned positions. Highly variable segments with insertions/deletions in many sequences were excluded from the analysis, leaving 60 well-aligned positions within N-terminal (in HXB2 numbering AA C119-C131), central (E153-I184), and C-terminal (Y191-C205) conserved elements.

Total beta-sheet or alpha-helix propensities for 5-residue segments within a window sliding along V1/V2 domain sequence were calculated:
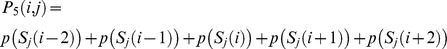



Thus, vectors of propensities across 106 sequences were generated for each position within the alignment of V1/V2 domains. Pierson correlation coefficient of log_10_ ID50 with total propensity and 5-residue segment propensities for each position was calculated. Randomization test [46] was used to estimate p-values of the correlations (1,000,000 samples). To control errors arising from multiple hypothesis testing, positive false discovery rate (pFDR) approach of Storey [47] was applied. Estimated pFDR for beta-sheet propensity correlations across 60 positions at 0.05 p-value cutoff was 13%.

## Supporting Information

Figure S1 Plots of Pierson correlation coefficients R and p-values for the AHP/NS correlation. Plots are made the same way as for BSP on Figure 4.
(TIFF)
