# Supplementary figures and images for: Estimated Secondary Structure Propensities within V1/V2 Region of HIV gp120 Are an Important Global Antibody Neutralization Sensitivity Determinant
Source: PLoS One. 2014 Apr 4;9(4):e94002. doi: 10.1371/journal.pone.0094002 (PMC3976368; doi:10.1371/journal.pone.0094002)

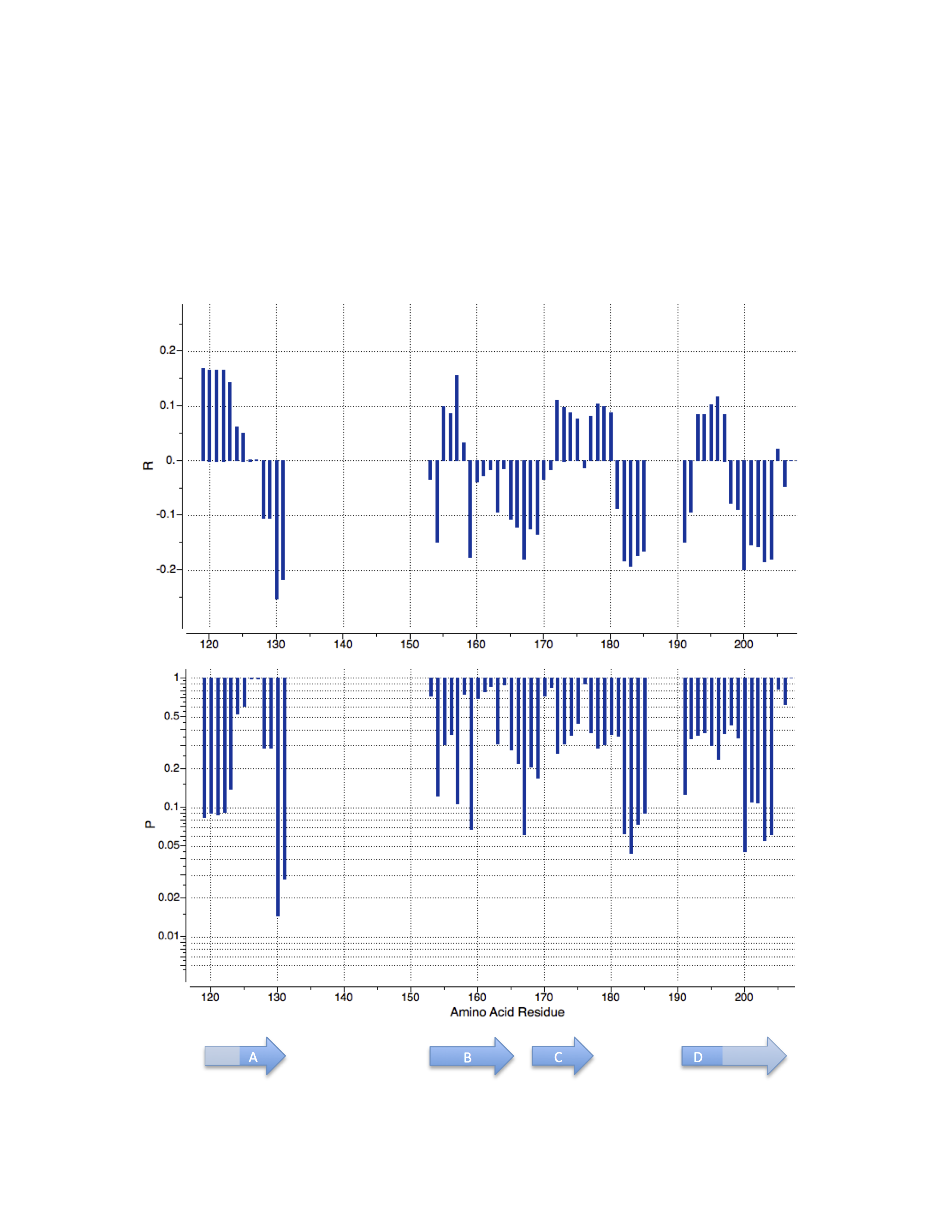

Supplement: Figure S1 — Plots of Pierson correlation coefficients R and p-values for the AHP/NS correlation. Plots are made the same way as for BSP on Figure 4. (TIFF) [file pone.0094002.s001.tiff]
